# THE IMPACTS OF DEPRESSION AND SUICIDE ATTEMPTS AMONG OLDER MEN WHO HAVE SEX WITH MEN

**DOI:** 10.1093/geroni/igac059.3033

**Published:** 2022-12-20

**Authors:** Alex Siu, Wing Chan, Elsie Yan

**Affiliations:** Hong Kong Polytechnic University, Hong Kong, Hong Kong; Hong Kong Polytechnic University, Hong Kong, Hong Kong; The Hong Kong Polytechnic University, Hong Kong, Hong Kong

## Abstract

Suicidality among older adults has attracted much attention due to their vulnerability. Older men who have sex with men (OMSM) have rarely been studied psychologically. Study examines factors that affect the mental health of OMSM, including depression, suicidal tendency, and suicide likelihood. OMSM in the United States are analyzed using descriptive statistics for correlations between depression and suicidal tendency. A literature review helped us select scales based on the regression model we constructed. Control variables were assessed for validity and relevance. A dependent variable was depression, and a dependent variable was suicidal tendencies. Depression and suicidal tendency scores significantly differed between men who have sex with men and the general population (t = 67.084,58.193, P < 0.01). Suicidal tendencies and depression are significantly higher among homosexuals than among general groups. The regression analysis shows older men who have sex with men are more likely to suffer from depression and suicide (P < 0.01). Depression and suicide rates in OMSM are higher than those in the general population. The level of depression, in the intermediary test, mediates both the effect of OMSM on individual suicidal tendency and individual suicidal behavior (P < 0.01). Suicidal tendencies in OMSM can be reduced through depression intervention.

